# Visualization of the Sol–Gel Transition in Porous Networks Using Fluorescent Viscosity-Sensitive Probes

**DOI:** 10.1021/acs.jpclett.3c02634

**Published:** 2024-01-11

**Authors:** Romane Le Dizès Castell, Elham Mirzahossein, Marion Grzelka, Sara Jabbari-Farouji, Daniel Bonn, Noushine Shahidzadeh

**Affiliations:** † Institute of Physics, University of Amsterdam, Amsterdam 1098XH, The Netherlands; ‡ Laboratoire Léon Brillouin, Université Paris-Saclay, CEA-Saclay, 91191 Gif-sur-Yvette Cedex, France

## Abstract

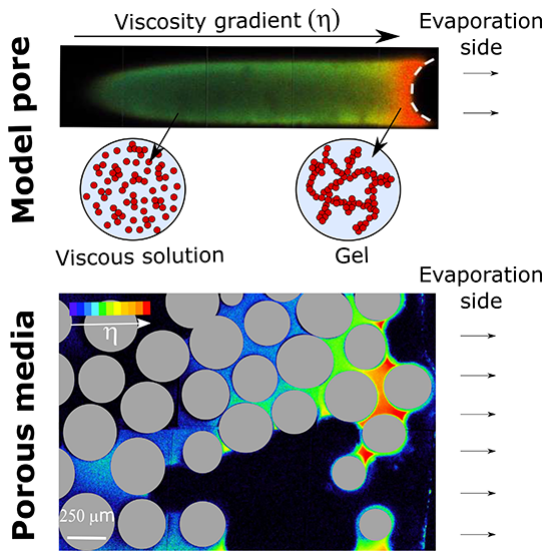

The sol–gel transition involves the transformation of a colloidal suspension into a system-spanning, interconnected gel. This process is widely used to reinforce mechanically weakened porous artifacts, such as sculptures but the impact of the restricted geometry (porous network) on the gelation dynamics of the solution remains unclear. Here, using fluorescent viscosity-sensitive molecular rotors, confocal microscopy, and model pores, we visualize the local viscosity changes at the microscale that accompany the sol–gel transition of a methyltriethoxysilane solution into a gel network. We show that, with evaporation of the solvent, a viscosity gradient develops near the free surface, triggering the sol–gel transition inside small pores near the surface. In homogeneous porous media, this leads to skin formation, which reduces the evaporation rate. In heterogeneous porous media, a gradient in gel density develops toward the heart of the porous material, where the gel formation mainly occurs as capillary bridges within smaller pores.

The sol–gel process is extensively used to produce a wide range of materials including coatings, porous glasses for sensors, and optical components as well as more recently to consolidate porous materials for cultural heritage.^1−5^ In the latter application, after injection of a solution in the porous medium and by evaporation of the solvent, the formation of a thin layer of gel inside the porous network mechanically strengthens the degrading stone. When a porous material is saturated with a fluid, the latter will subsequently flow in the restricted geometry of pores connected by throats at the micro/mesoscale. Such confinement at small scales can change the fluid properties because of geometric constraints and capillary pressure development. The properties of the porous medium such as pore size distribution, porosity, surface area, or the chemical nature of the interface define and can thus largely affect the liquid dynamical behavior during flow induced by evaporation, imbibition, or drainage. In general, investigating the kinetics of such phenomena in porous materials is quite complex, as it requires the combination of different advanced imaging techniques, such as nuclear magnetic resonance (NMR) to visualize the saturation profiles of the liquid and X-ray microtomography to visualize microscale changes in the porous matrix such as salt precipitation or colloidal deposits. In this way, previous investigations of drying of colloidal suspensions and ionic solutions in stones have revealed accumulation of particles^6,7^ or salts^8^ at the evaporative surface of the porous media, not unlike the famous “coffee stain effect” for evaporating drops.^9^ However, the dynamics of gelation in confinement is still poorly understood as multiple physical phenomena will occur during liquid flow and phase change, including a viscosity increase and mass transport due to evaporation of the solvent. Here, by employing fluorescent molecular rotors and confocal microscopy, we probe the local viscosity changes and follow the dynamics of gelation in microconfinements representative of porous networks in real porous materials. We are therefore able to relate the drying kinetics to the physical properties of the solutions over time, especially the local flow behavior and its consequences.

Fluorescent molecular rotors have previously been used as viscosity sensors for biological samples,^10−12^ to probe at different scales the structure and properties of fluids such as polymer solutions^13−15^ and polymer glasses^16,17^ and to study the aging of gels.^18−20^ However, in these previous investigations, a correlation was sought between the macroscopic mechanical properties and the fluorescence of the molecular rotor. To the best of our knowledge, such viscosity-sensitive probes have not yet been used to monitor the spatial development of a gel at the microscale in a porous network. Our results reveal that, contrary to what is commonly expected, the sol–gel transition does not occur in a homogeneous way in the whole sample and is also strongly dependent on the pore size distribution of the porous matrix. This can lead either to a gradient of gel deposited preferentially in the small pores from the free surface to the heart of the materials or to the formation of a skin, which can drastically reduce the evaporation of liquids from the porous materials later on.

Here, we investigate the sol–gel transition of a methyltriethoxysilane (MTEOS) solution during evaporation. This transition is known to occur in three stages:^21^ (1) polymerization of hydrolyzed MTEOS monomers to form macromolecular gel precursors, (2) growth of the precursors, and (3) linking of macromolecules into a system-spanning network. The last step is accelerated by the evaporation of the solvent and characterized by an abrupt rise of the viscosity and the appearance of an elastic response to stress^22^ (see Supporting Information).

To study the gelation dynamics, we used 4-[4-(dimethylamino)styryl]-1-methylpyridinium iodide (4-DASPI) as a fluorescence probe. 4-DASPI is highly sensitive to variations in viscosity within its molecular environment. When the probe is photoexcited in a low-viscosity environment, the torsion of the aniline moiety activates a nonradiative pathway, resulting in a significant decrease in the fluorescence intensity and lifetime of the probe.^23^ When the internal rotation is impeded by molecular crowding in the microenvironment, the nonradiative pathway is deactivated and photon emission occurs, corresponding to an increase in the fluorescence intensity and lifetime of the probe.

As model porous media, we use microcapillaries and home-built quasi-2D porous media with interconnected pores.^24^ Such microfluidic model systems have been recently extensively used to follow in real time under the optical microscope the dynamics of the phase transition^25,26^ and transport processes at the pore scale in confinement.^27−30^ Capillaries are also the elementary units in the pore networks models^31,32^ and provide the input for the development of lattice Boltzman simulations for drying with complex fluids.^33^

First, we study the rheology of drops of hydrolyzed MTEOS solution with small concentrations of 4-DASPI (Experimental Methods). The results for the storage modulus (*G′*) and the loss modulus (*G′*), along with the fluorescent intensity results, are plotted in Figure 1a. The sharp increase of the storage modulus *G′* at *t* ≈ 1000 min reflects the rapid formation of clusters and percolation of the macromolecules in the sample. This process carries on until the storage modulus becomes equal to the loss modulus, thus defining the sol–gel point (*t* ≈ 1440 min in Figure 1a).^34^ Note that the maximum value of the fluorescence intensity of 4-DASPI is reached in the vicinity of the sol–gel point, confirming that 4-DASPI is an adequate probe to optically monitor the sol–gel transition of MTEOS. However, a small decrease in fluorescence intensity, due to the effect of photobleaching, can still be observed after the sol–gel point (∼10%).

**Figure 1:**
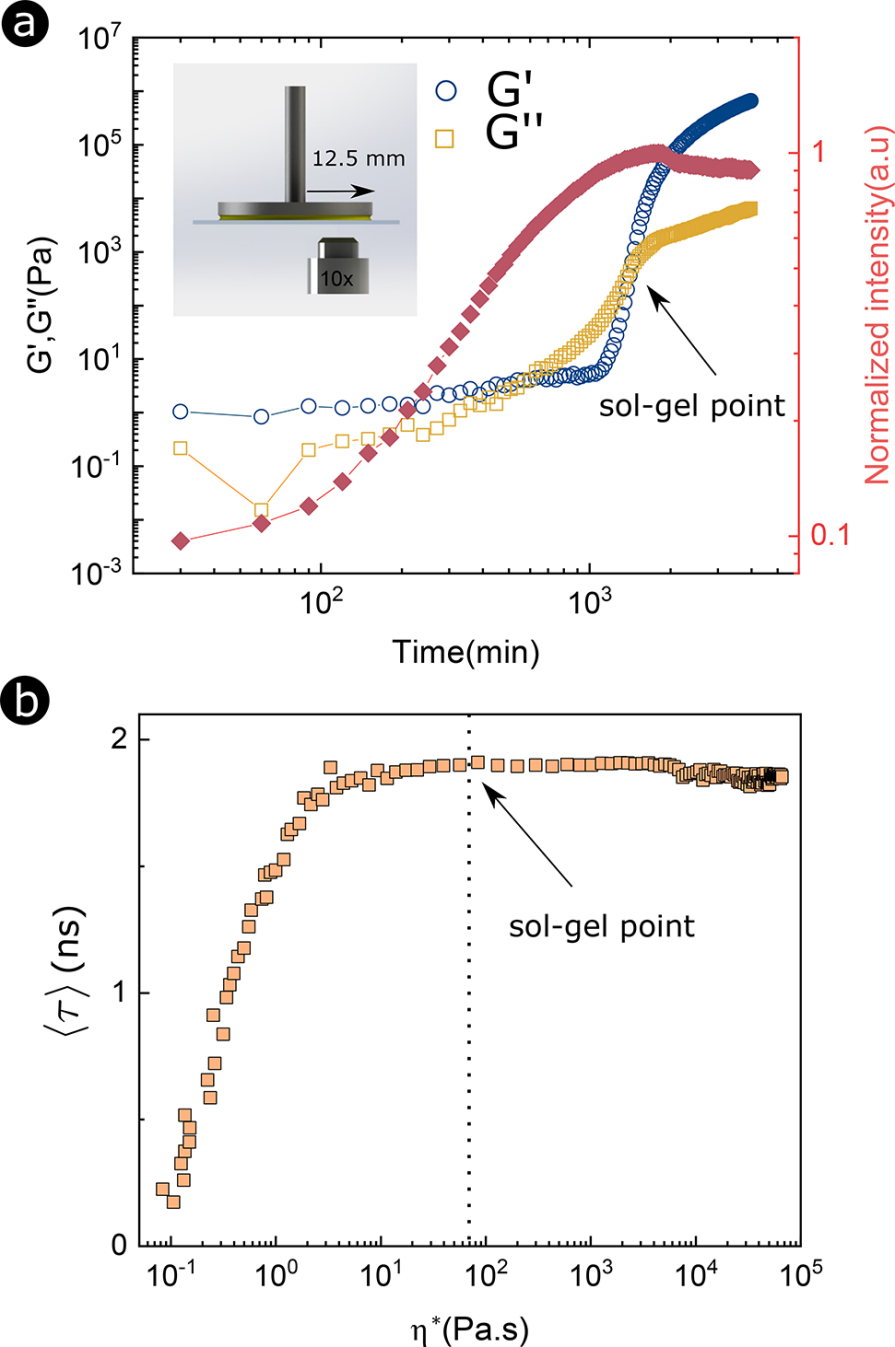
Sol–gel transition of MTEOS induced by evaporation. (a) Time evolution of the storage *G′* (blue circles) and loss *G″* (yellow squares) moduli as well as fluorescence intensity of the probe (red squares) during the sol–gel transition. (b) Amplitude-averaged fluorescence lifetime ⟨τ⟩ as a function of the magnitude of the complex viscosity |η*|.

Fluorescent lifetime imaging microscopy (FLIM, Figure 1b) shows that the fluorescence intensity values directly correlate with the amplitude-averaged fluorescence lifetime ⟨τ⟩, see SI. We can therefore plot the fluorescence lifetime of the probe as a function of complex viscosity defined as  (with ω being the frequency of oscillation) at each time step in Figure 1b. From this figure, we estimate that the sol–gel point occurs at a viscosity of |η*| = 83 Pa·s, corresponding to a fluorescence lifetime of ⟨τ⟩ = 1.9 ns. We used this value as a threshold for identifying the gelation point in microscopic scale experiments. As the intensity, lifetime, and viscosity are proportional to each other, we will interchange these terms in the following analysis. We nonetheless prefer FLIM in the experiments as, contrary to intensity measurements, the fluorescence lifetime is independent of the probe’s concentration and the effects of photobleaching.

We subsequently use confocal microscopy to investigate the sol–gel transition in confinement with round and square capillaries as pore models (see Experimental Methods). In Figure 2a, fluorescence lifetime images of the gelation process in round capillaries at different times are shown, from which we calculate the volume evolution of the MTEOS solution during gelation (Figure 2b). During the first stage of the drying process (Regime 1 in Figure 2b), the meniscus gradually recedes in the capillary. The lifetime of 4-DASPI fluorescence—and thus the solution’s viscosity—increases slowly and homogeneously. In this regime, the loss of volume during drying follows a square root of time as described by Stefan^35^ for a diffusive process. When the MTEOS is almost completely dried, the kinetics deviates from a square root of time (Regime 2 in Figure 2b). In Figure 2a, this corresponds to the formation of a gradient in viscosity from the evaporative meniscus to the bulk of the solution (*t* = 660 min in Figure 2a). The solution starts to gelate at the evaporative meniscus, and a skin forms between 600 and 900 min (see *t* = 1080 min in Figure 2a). From there on, the drying kinetics slows down significantly. Moreover, Figure 2c depicts the local viscosity, calculated from the surface of the meniscus extending into the bulk (distance *d*) over different time steps. It reveals that the gradient of viscosity intensifies over time, primarily due to the gelation of the meniscus surface (skin formation) occurring first. Such skin formation is well-known for polymer solutions or colloidal suspensions^14,27,37−40^ where a significant decrease in the evaporation rate has also been observed.^27,38^

**Figure 2:**
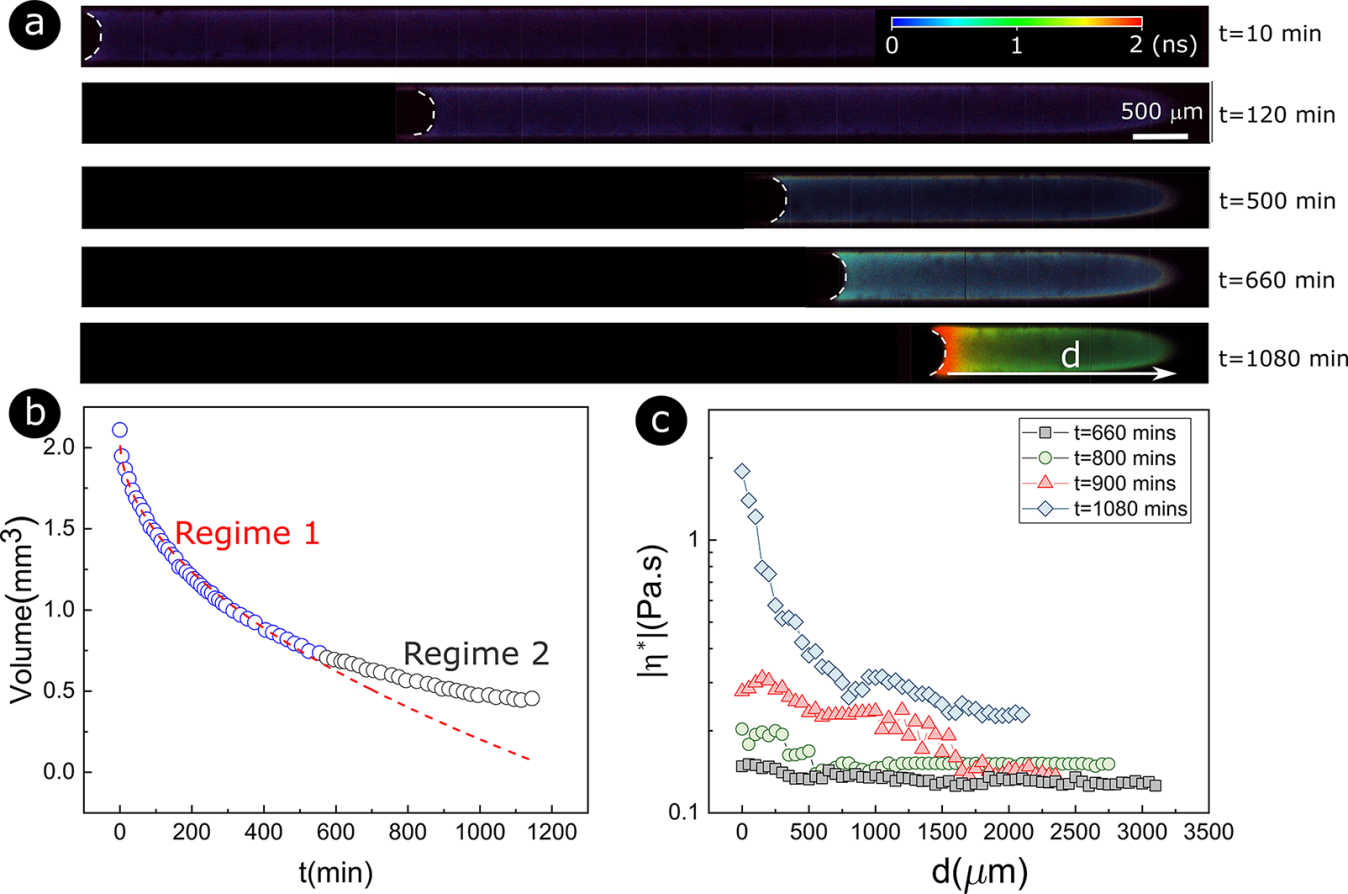
Sol–gel transition of MTEOS in a round capillary open only on the left side. (a) FLIM of the gelation process at different evaporation times. (b) MTEOS volume change as a function of evaporation time, as calculated from images such as in (a). Initially, the volume changes can be described by *V* = −0.59*t*^0.5^ + 2.068 (dashed red line). (c) Calculated viscosity profiles derived from fluorescence lifetime measurements (SI) as a function of distance *d* from the surface of the meniscus.

Square capillaries are generally considered to be more representative of a real pore due to the presence of corners and angles. Comparing the fluorescence lifetime images of Figures 2 and 3, we find that the drying process in square capillaries is very different from that in round capillaries. Liquid flows are formed in the corners by capillarity, and the viscosity starts to increase first at the tip of those corner flows. As drying proceeds, the corner flows become highly viscous on an increasing length until the moment at which the corner flows reach the maximum viscosity and the bulk meniscus starts to gelate.To understand this local gelation at the tip on the corner, one should consider the displacement of the MTEOS macromolecules in the fluid with evaporation. Originally, the sol in the capillary is composed of macromolecules homogeneously dispersed within the liquid in the capillary. As evaporation proceeds, liquid flows in the corners and the macromolecules can be advected to the surface, similarly to the coffee ring effect observed in droplets.^41^ The advection of macromolecules to the surface is generally counterbalanced by the diffusion process, which tends to equilibrate and homogenize the concentration gradient inside the fluid. The competition between the two processes can be quantified by Peclet number, which gives the ratio of the diffusion time τ_*diff*_ = *L*^2^/*D* to the advection time τ_*adv*_ = *L*/*v*, resulting in
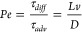
1where *L* is the length scale of the corner flows, *v* is the velocity of the fluid flow in the corners, and *D* is the diffusion rate of the MTEOS macromolecules in the fluid. Here, in every corner of the capillary,  with , and *S* and *V* represent the evaporation surface and the evaporated volume obtained from image analysis, respectively. *D* in eq 1 can be estimated from the Stokes–Einstein law at the early stages of drying:
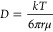
2with the radius of the macromolecules *r* = 3 nm (see Figure S1(21,42)) and μ the viscosity of the solution. Knowing that at the beginning of the drying (*t* = 10 min), μ = 5 × 10^–3^ Pa·s, *L* = 1.95 mm, and *v* ≈ 9 × 10^–4^ mm·s^–1^, we find that *Pe* ≈ 80 > 1. The macromolecules are thus advected at the tip of the corner flows, resulting in a high concentration of macromolecules in that region. This high concentration leads to local gelation, causing a significant increase in viscosity and affecting the overall drying kinetics. Indeed, as visible in Figure 3b, in the first drying period, the drying kinetics follows a square root of time, contrary to what is commonly observed in square capillaries where the advection of the liquid at the surface by corner flows leads to constant-rate evaporation.^32,43^ During this initial period, the substantial increase in viscosity impedes further evaporation from the tip of the corner flows. As a result, water evaporation occurs below the gelation front inside the capillary. A second regime is observed once the corner flows are fully gelated, characterized by a slower volume change. It should be emphasized that, although the drying kinetics is similar for round and square capillaries, the gelation process is quite different: the square capillary is fully gelated after *t* = 1080 min, whereas the round capillary is still not fully gelated even after *t* = 3070 min.

**Figure 3:**
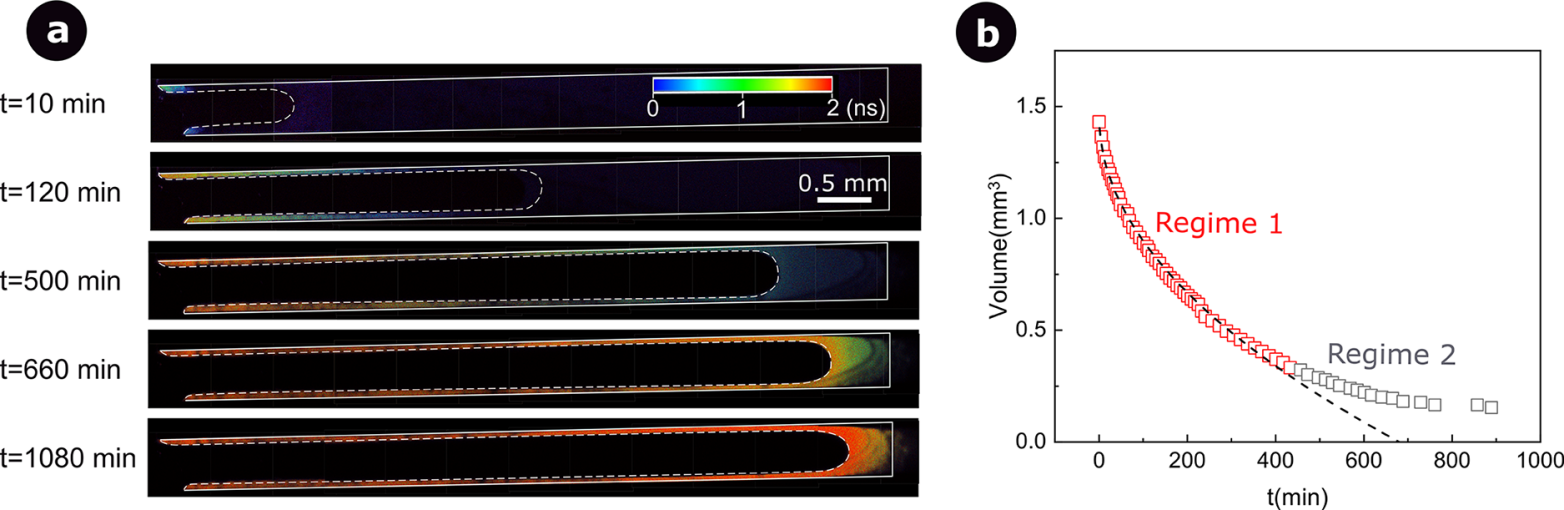
Sol–gel transition of MTEOS in a square microcapillary open only on the left side. (a) FLIM images of the gelation process at different time points. (b) MTEOS volume change as a function of evaporation time, as calculated from images such as those in (a). At first (regime 1), the volume change can be described by the equation *V* = −0.56*t*^0.5^ + 1.46 (black dashed line).

Of course, our model capillaries cannot fully grasp the complexity of the drying processes in real porous media, where the liquid can flow by capillarity between pores of different sizes. Therefore, we turn to quasi-2D model porous media, which provide a more realistic description of real-world porous media like stones. We designed quasi-2D micromodels of porous media with monomodal or bimodal pore size distribution following the protocol detailed in the SI and adapted from ref (24). Bright-field microscope pictures of two such models are shown in Figure 4a (homogeneous porous medium with a monomodal pore size distribution) and Figure 4b (heterogeneous porous medium with a bimodal pore size distribution). The porosities ϕ of the homogeneous and heterogeneous porous media are determined using image analysis:
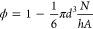
3where *A* is the area of the capillary, *h* = 0.3 mm is its width, and *N* is the number of sintered beads of diameter *d* = 250 μm. The resulting porosities are ϕ = 42% for the homogeneous porous medium in Figure 4a and ϕ = 50.1% for the heterogeneous porous medium in Figure 4b. These values are close to the porosities observed in some sedimentary stones such as Maastricht limestone or Mesne sandstone.^44^

**Figure 4:**
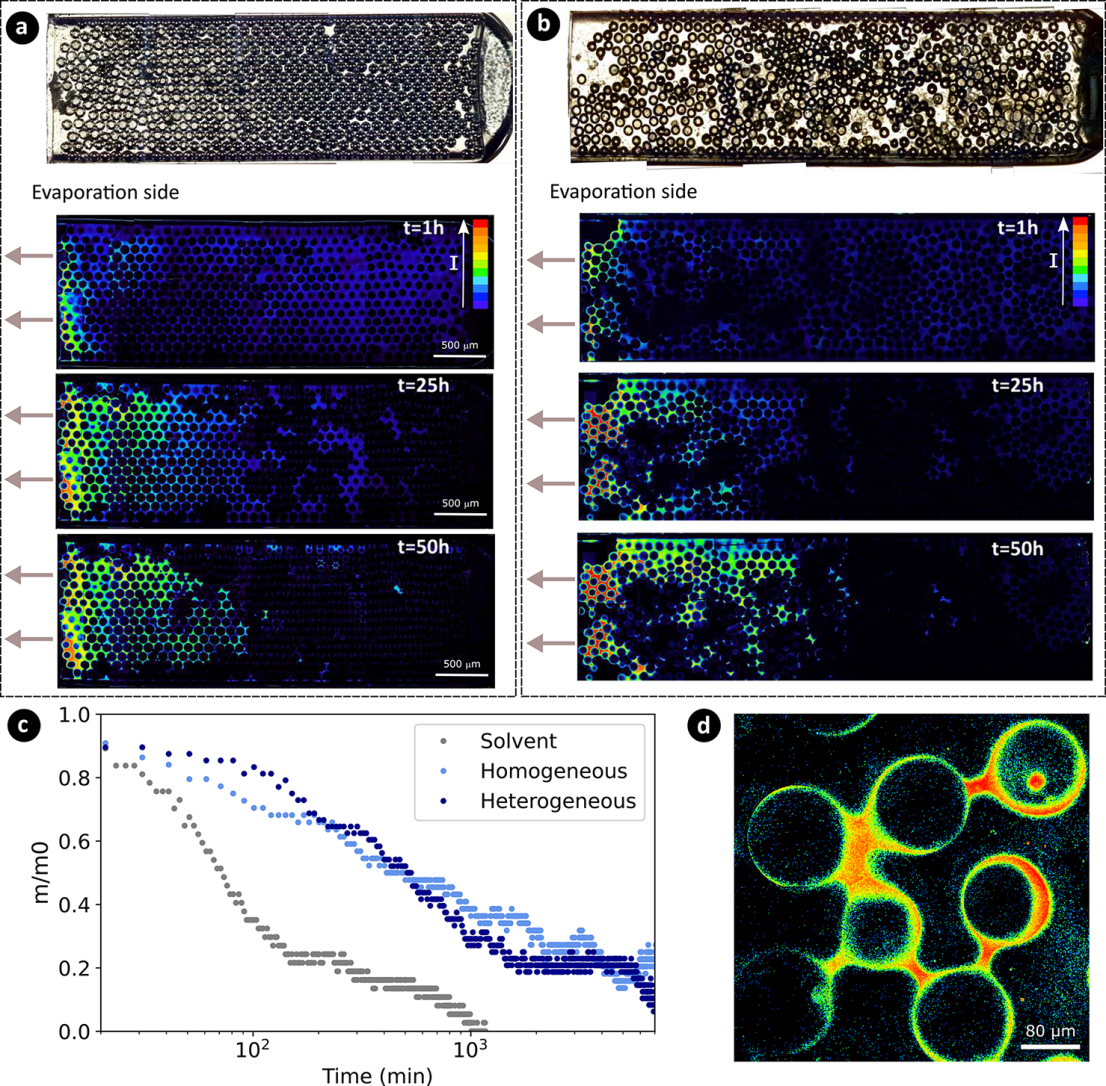
Sol–gel transition of MTEOS induced by evaporation. (B) MTEOS induced by evaporation. Top: optical microscopy pictures of empty 2D model homogeneous (a) and heterogeneous (b) porous media. Bottom: confocal microscopy snapshots at *t* = 1, 25, and 50 h during the sol–gel transition. The red areas in fluorescence images represent the gel state, while the green areas represent highly viscous liquid. (c) Mass of the solution inside the homogeneous and heterogeneous 2D porous media, relative to the initial mass at the start of the drying process, as a function of evaporation time. The gray symbols depict the evaporation of the solvent in the homogeneous 2D porous medium. (d) Confocal microscopy snapshot of gel capillary bridges between glass beads at the end of the drying.

Experiments are performed in both types of quasi-2D porous media by filling the capillary with MTEOS and 4-DASPI solutions (same concentration as before). The gelation dynamics are monitored with confocal microscopy (Figure 4a,b for homogeneous and heterogeneous porous media, respectively). We used the aforementioned fluorescence lifetime threshold (1.9 ns) to determine the full gelation in the sample (red regions in Figure 4). One can see that in both cases, the viscosity of the solution increases mostly at the evaporation side as the porous media desaturates. At the end of the experiment (*t* = 50 h in Figure 4), the gel is not homogeneously dispersed within the porous media but is mainly localized at the entrance. For the heterogeneous porous medium, the big pores are emptying first as the solvent evaporates. When it is dried, the gel is only localized at the entrance and in the small pores, forming capillary bridges (see Figure 4d). Heterogeneous distribution of the pore size strongly affects the distribution of gel within a porous medium because the capillary pressure in a pore of radius *r* is given by *P*_*c*_ = 2γcos θ/*r* where γ and θ are the surface tension and contact angle of the evaporating fluid. Due to a lower capillary pressure at larger pores, liquid flows from big pores to small pores as the porous medium desaturates with evaporation.^45^ The small pores thus remain filled for a longer time. Similar to the case of square capillaries, the liquid flows in small pores can lead to the preferential advection of macromolecules, and one can estimate the Peclet number from eq 1 for the macromolecules in the liquid at the initial stage of the drying (i.e., when the viscosity is equal to the initial viscosity). For the length of the homogeneous porous media *L* = 11 mm and evaporation speed *v*_*evap*_ = 1 × 10^–6^ m·s^–1^, we find *Pe* ≈ 800, showing that the macromolecules are indeed advected at the evaporation surface at the beginning of the drying. Due to their high concentration, the gelation starts there in a very short time as visible from the intensity values (i.e., viscosity here) in Figure 4.

In Figure 4c, we show how the relative weight decreases with time for both types of model porous media. The drying dynamics is similar for homogeneous and heterogeneous porous media, even though there are strong differences in the distribution of gel in the two porous media. For both, gelation at the surface results in a much slower evaporation compared to a solvent-only scenario, right from the onset of the drying process. Similar results were reported for particle deposition in porous media.^6,7^ In porous media, the velocity of liquid flows by capillarity at the surface *v*_*cap*_ can be approximated by Darcy’s law  where μ is the viscosity of the liquid, *k* the permeability of the network, and  the capillary pressures in the pores network, function of the contact angle, and surface tension of the solution. We show in the SI that both the variation of the contact angle and the surface tension of the solution are insignificant compared to the increase in viscosity. Thus, during the sol–gel transition, as the viscosity increases locally at the surface, *v*_*cap*_ decreases accordingly until the evaporation velocity  exceeds *v*_*cap*_, the velocity at which the liquid reaches the evaporation surface. From that point onward, the solution continues to evaporate inside the porous media, and the vapor diffuses over an increasing length scale. Moreover, according to our results with round capillaries, we can assume that once the gel is formed, the evaporation from the gelified region through the nanopores of the gel is negligible. The decrease in the drying rate can therefore be explained by the formation of a drying front due to the high viscosity of the solution near the evaporation side. Such a receding front, while not visible in those experiments, has already been observed by some of the coauthors^46^ in the context of evaporation-induced sol–gel transition of MTEOS in sandstones.

In conclusion, we studied the drying of a sol–gel solution inside models of single pores and porous media. We determined the local viscosity using fluorescent molecular probes and related the outcomes to the decrease in the evaporation rate in single pores and 2D porous media with interconnected pores. By comparing round and square capillaries, this technique allowed us to reveal the impact of corner flows on the gelation kinetics and on the final distribution of the gel. In the 2D model porous media, we observed that a heterogeneous gelation front appears near the evaporation surface due to the advection of macromolecules. Our study reveals that a gradient of gel develops, starting from the evaporative boundary and next invading the porous media, accompanied by a sharp decrease in the evaporation rate. Moreover, we show that local fluorescent viscosity probes are a promising new method to study the drying and transport of complex fluids in porous media without the need for highly advanced imaging techniques such as NMR or X-ray microtomography. Our results shine new light onto the sol–gel transition in confinement and the dynamics of gel formation in a porous network. This is of great interest for the conservation of our stones’ cultural heritage, as silanes are used very frequently as consolidants.^1,4,5^ The results described here show the formation of a thin film of gel, which is fairly heterogeneous and could affect the materials’ dynamics and their aging. For example, the skin effect in homogeneous porous stone may lead to waterproofing, making it difficult to “breathe” or dry out after sol–gel treatment. It should therefore be used with caution, contrary to heterogeneous porous materials, where the larger pores will remain permeable, allowing the flow of vapor and liquids. This study nonetheless suggests that in cracked and weathered porous materials, the gel would form along the cracks, where the evaporation rate is higher and could in this way prevent or delay the propagation of the crack’s tip. In addition, decreasing the Peclet number by applying a treatment at high relative humidity could be another alternative to achieve a more homogeneous consolidation.

## Experimental Methods

We used 4-[4-(dimethylamino)styryl]-1-methylpyridinium iodide (4-DASPI, from Sigma-Aldrich) as a fluorescence probe to monitor the dynamics of the sol–gel transition of a methyltriethoxysilane solution (MTEOS, from Sigma-Aldrich). The MTEOS solution is hydrolyzed prior to its use (see SI) to form a solution of methylsilanetriol (if complete hydrolysis). For the sake of simplicity, we use the term MTEOS to refer to the hydrolyzed solution in the rest of the Letter. When the pH during the hydrolysis and the condensation is below 7, the initial sol is composed of macromolecules in suspension of radius of 2–4 nm (see dynamic light scattering measurement in the SI and ref (42)). Evaporation of the solvent and condensation reactions afterward trigger the formation of the gel network.

Macrorheology measurements are performed on a drop of MTEOS containing a concentration of 10^–6^ mol L^–1^ 4-DASPI. The drop is deposited on a microscope cover slide. During the gelation, a rheometer head (Anton Paar, DSR502) is mounted on top of a confocal microscope (Zeiss Axiovert 200 M); see the inset of Figure 1(a). The drying experiment takes place at relative humidity *RH* = 45% and temperature *T* = 21 °C. The macrorheology is measured using a 25 mm diameter top plate that together with the bottom microscope slide constitutes a plate–plate geometry. The oscillatory shear experiments are done at a frequency of 10 rad/s with a small strain amplitude of 0.25% to measure the linear viscoelastic response; it was verified that neither the frequency nor the amplitude alters the response. The fluorescence intensity of 4-DASPI is measured locally with the confocal microscope underneath; we find that the gelation starts at the edges and then continues toward the center of the plate. For calibration, we measure the fluorescence intensity (or, equivalently, the fluorescence lifetime) near the meniscus formed at the edge of the geometry. We do this because by far the largest contribution to the viscoelastic properties that the rheometer measures comes from the edge of the sample; in this way, we can calibrate.

To investigate the sol–gel transition in confinement, we used single capillaries as models for basic units of pores. Experiments are performed in round (Vitrocom, inner diameter 50 μm) and square (Vitrocom, inner dimension 500 μm) capillaries, each closed on one side. In this study, quasi-2D micromodels of porous media with monomodal or bimodal pore size distribution are also designed, following the protocol detailed in the SI and adapted from ref (24). The capillaries and quasi-2D micromodels are filled with the solution of MTEOS and 4-DASPI (same concentration as before), and the drying and gelation are monitored with confocal microscopy. The time-resolved fluorescence decay was assessed using time-correlated single photon counting (TCSPC) on an inverted confocal microscope (Leica, TCS SP8). The excitation wavelength employed was 470 nm, with the emission spectrum recorded between 500 to 700 nm. As the temperature change due to evaporative cooling during the evaporation of the solvent is expected to be less than 5 K,^47,48^ the influence of the temperature change on the fluorescence properties of 4-DASPI can be neglected.^49^

## Supporting Information Available

(1) Chemical reactions during the sol–gel transition of methyltriethoxysilane; (2) dynamic light scattering measurements during the sol–gel transition of MTEOS; (3) fluorescence properties of 4-DASPI inside MTEOS solution; (4) sol–gel transition of MTEOS droplets using time-resolved fluorescence microscopy; (5) evolution of the surface tension and contact on glass capillary wall of MTEOS during the sol–gel transition; (6) more measurements about the skin formation in round capillaries; (7) protocol to manufacture 2D model porous media (PDF)
