# Supplementary material for: Visualization of the Sol–Gel Transition in Porous Networks Using Fluorescent Viscosity-Sensitive Probes
Source: J Phys Chem Lett. 2024 Jan 11;15(2):628–35. doi: 10.1021/acs.jpclett.3c02634 (PMC10801688; doi:10.1021/acs.jpclett.3c02634)
Supplement: Supplementary file 1 — jz3c02634_si_001.pdf [file jz3c02634_si_001.pdf]

Visualization of the Sol-Gel Transition in Porous Networks Using Fluorescent Viscosity-Sensitive Probes

Romane Le Dizès Castell,*,† Elham Mirzahossein,*,† Marion Grzelka,‡ Sara Jabbari-Farouji,† Daniel Bonn,† and Noushine Shahidzadeh†

†*Van der Waals-Zeeman Institute, Institute of Physics, University of Amsterdam, 1098XH Amsterdam, The Netherlands*

‡*Laboratoire Léon Brillouin, Université Paris-Saclay, CEA-Saclay, 91191 Gif-sur-Yvette Cedex, France.*

E-mail: r.ledizes2@uva.nl; e.mirzahossein@uva.nl

Supporting Information

The Supporting Information contains details on:

1. Chemical reactions during the sol-gel transition of methyltriethoxysilane,
2. Dynamic Light Scattering measurements during the sol-gel transition of MTEOS,
3. Fluorescence properties of 4-Daspi inside MTEOS solution,
4. Sol-gel transition of MTEOS droplets using time-resolved fluorescence microscopy,
5. Evolution of the surface tension and contact on glass capillary wall of MTEOS during the sol-gel transition,
6. More measurements about the skin formation in round capillaries,
7. Protocol to manufacture 2D model porous media.

1. Chemistry of sol-gel transition with methyltriethoxysilane

Methyltriethoxysilane $(C_2H_5O)_3SiCH_3$ (Sigma-Aldrich) is hydrolysed prior to its use by a 0.1 mol.L^{-1} solution of acetic acid (Sigma-Aldrich). The hydrolysis reaction is the following:

$$(CH_3CH_2O)_3SiCH_3 + 3\,H_2O \longrightarrow CH_3Si(OH)_3 + 3\,CH_3CH_2OH \qquad \{1\}$$

where the resulting solution consists $CH_3Si(OH)_3$ methylsilanetriol MTS in a solvent composed of water and ethanol (formed during the hydrolysis of the MTEOS). After (partial) hydrolysis, evaporation of the solvent triggers condensation reactions in the solution that lead to the formation of a highly branched gel network spanning through the whole sample.[1] Generally, condensation reactions between the MTS monomers formed start before the hydrolysis is complete. Two condensation reactions can describe the sol-gel process:

$$CH_3R'_2Si - OCH_2CH_3 + HO{-}SiCH_3R'_3 \longrightarrow CH_3R'_2Si - O - SiCH_3R'_3 + CH_3CH_2OH \qquad \{2\}$$

$$CH_3R'_4Si - OH + HO{-}SiCH_3R'_5 \longrightarrow CH_3R'_4Si - O - SiCH_3R'_5 + H_2O \qquad \{3\}$$

where R'_2, R'_3, R'_4 and R'_5 are methylsilanes at different condensation states.

2. DLS measurements

We used a Dynamic Light Scattering setup (ALV ALV/DLS/SLS-5000) to determine the particle size distribution with a wavelength of 633 nm at a 90°angle. Fig.S1 is the DLS measurement of three different stages of the sol-gel transition of MTEOS. After the hydrolysis

of the MTEOS, the solution consists of monomers with the size of 2-3 nm (Fig.S1a) in suspension in the solvent. When the evaporation starts, the monomers start to aggregate and form oligomers with a broader size distribution with the radius of 2-12 nm. Bigger clusters are also formed, with a size around 1-2 μm. This shows the creation of a second population of bigger oligomers (Fig.S1b). With further evaporation, oligomers polymerized and interlinked to form a gel network (Fig.S1c). At that stage, due to the limitation of the DLS setup and multi scattering effect during gelation, exact particle sizes is not reliable anymore.

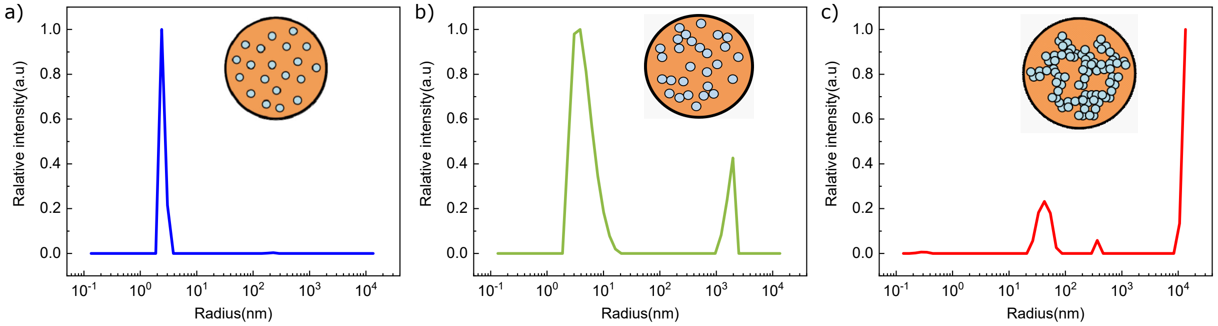

Figure 1: Dynamic light scattering(DLS) measurements: The Three stage scheme of the sol-gel transition. a) Formation of the MTS monomers after the hydrolysis process b) Formation of the oligomers c) Growth of the clusters and percolation formation

3.Fluorescence properties of 4-Daspi inside MTEOS solution

Here we look at the emission spectrum of 4-Daspi inside the MTEOS solution and gel for the excitation wavelength of 470nm. We observe stokes shift of the emission spectrum after gelation. The emission span that we are measuring in FLIM is between 500 to 700 nm. The absorbance spectra exhibit a peak at approximately 470 nm in the solution state and 488 nm in the gel state. The absorbance spectra are notably small since the concentration

of 4-Daspi is low (C= 10^{-6}mol.L^{-1}) inside the sample. It is important to note that the measured fluorescence lifetime is independent of concentration and the precise absorption and emission wavelength.

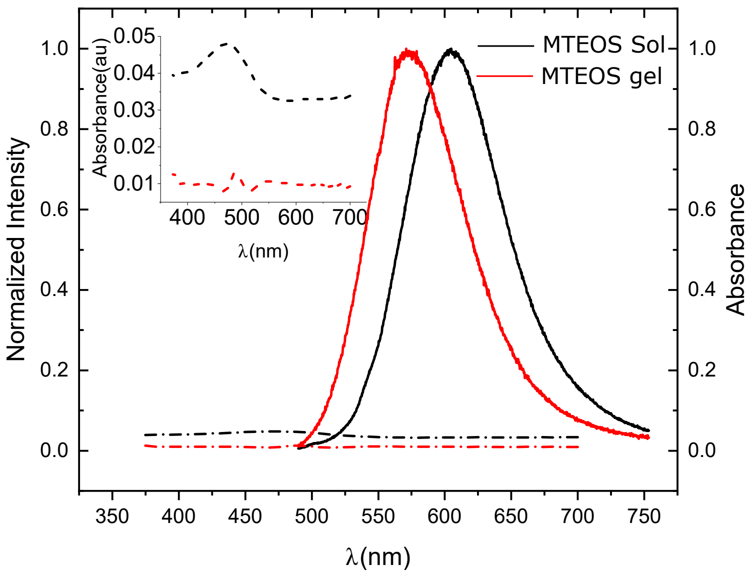

Figure 2: Emission and absorbance spectra of 4-Daspi inside MTEOS solution before and after gelation. The emission spectrum is measured for an excitation wavelength of 470nm. The solid line is the emission spectrum and the dashed line is the absorption spectrum.

4. The sol-gel transition of MTEOS droplet using time-resolved fluorescence microscopy

Time resolved fluorescence decay was measured by time-correlated single photon counting (TCSPC) on an inverted confocal microscope (Leica, TCS SP8) with a 20x dry objective (NA = 0.75) and a 56.6 μm pinhole. TCSPC's histogram is bi-exponential for the measurements reported, in which A_1 and τ_1 are the amplitude and lifetime of the first decay, and A_2 and τ_2 is the amplitude and lifetime of the second decay, respectively. All the values that are reported as a lifetime are the amplitude average lifetime $\langle\tau\rangle$ defined as:

$$\langle\tau\rangle = \frac{A_1\tau_1 + A_2\tau_2}{A_1 + A_2} \tag{1}$$

Which is in this case proportional to the steady-state intensity.

A general increase of amplitude averaged fluorescence lifetime $\langle\tau\rangle$ is observed as the gelation process proceeds.(FigS2.c.) Initially, $\langle\tau\rangle$ increases very slowly (almost constant) as evaporation occurs. Monomers are the most numerous species at the beginning. This follows up by a fast increase in the fluorescence lifetime which is related to the formation of bigger oligomers and cluster growth. In the last stage of the aging process, $\langle\tau\rangle$ increases with a smaller slope. This must occur after the gel network is created and the water pool inside the pores starts to slowly evaporate.

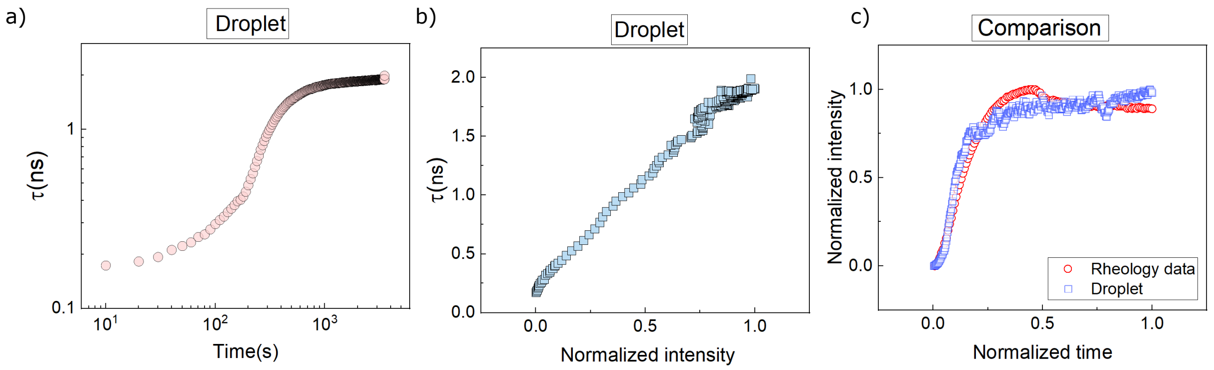

Figure 3: Fluorescence lifetime measurement of MTEOS droplet+4-DASPI: a) fluorescence lifetime as a function of time at the center of the droplet b) fluorescence lifetime as a function of intensity

The fluorescence intensity of the MTEOS solution is measured during gelation using a Zeiss microscope (Zeiss Axiovert 200 M) simultaneously with the Rheological measurement, as explained in the main text (Fig .1). Additionally, we use the FLIM setup of a Leica confocal microscope (Leica SP8) to study the gelation process of the MTEOS droplet by measuring fluorescence lifetime over time, as depicted in Fig.S2a. Through the FLIM setup, we directly measure the correlation between the fluorescence lifetime and fluorescence intensity of the fluorophore during gelation (Fig.S2b). Furthermore, a comparison of the fluorescence in-

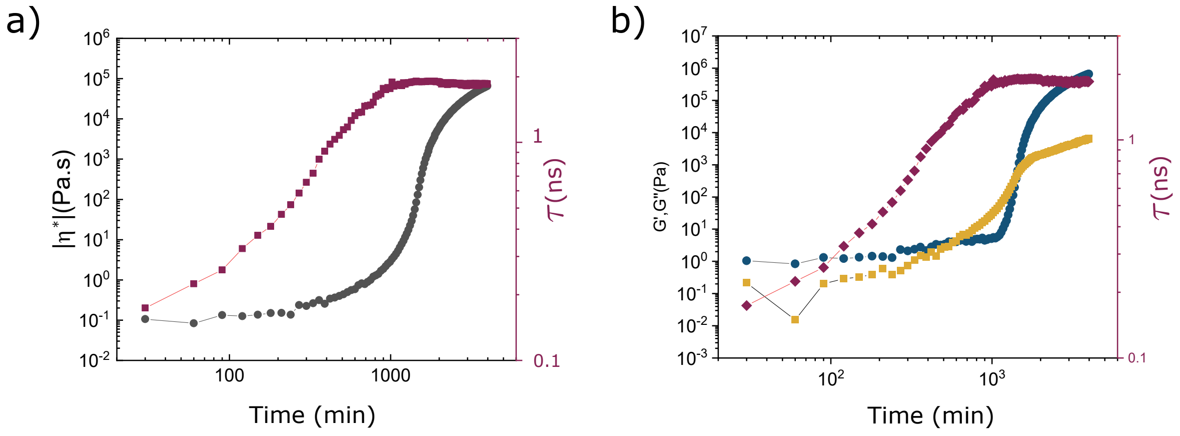

Figure 4: Rheological evolution of the sol-gel process

tensity between both microscopes is also conducted (Fig.S2c). By using (Fig.S2c) and the present linear relationship between lifetime and intensity in these measurements, we are able to establish a correlation between all the intensity values obtained from the Zeiss microscope and a corresponding fluorescence lifetime value.

5. Evolution of the surface tension and contact on glass capillary wall of MTEOS during the sol-gel transition

The variation of the surface tension of the solution during the sol-gel transition is measured with a Drop Shape Analyser from Kruss using the pendant drop method. The results are reported on Fig 5. As the gelation proceeds, a decreases in the the surface tension from 28 $mN.m^{-1}$ to 24 $mN.m^{-1}$ is observed.

The contact angle of the MTEOS solution on glass substrates is also likely to evolve during the sol-gel transition. But the initial solution is almost perfeclty wetting glass which makes it difficult to measure the contact angles. The contact angles were thus roughly estimated from the experiments performed in round capillary, see Fig.6. As visible in Fig.6,

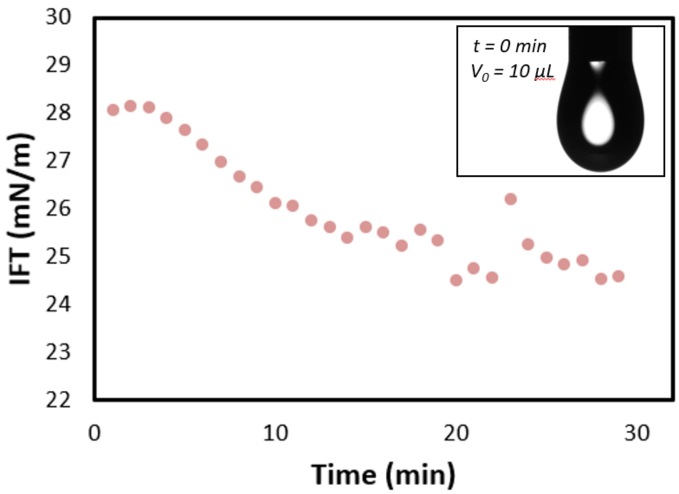

Figure 5: Surface tension evolution during the sol-gel transition of a 10 μL MTEOS droplet plotted as a function of time

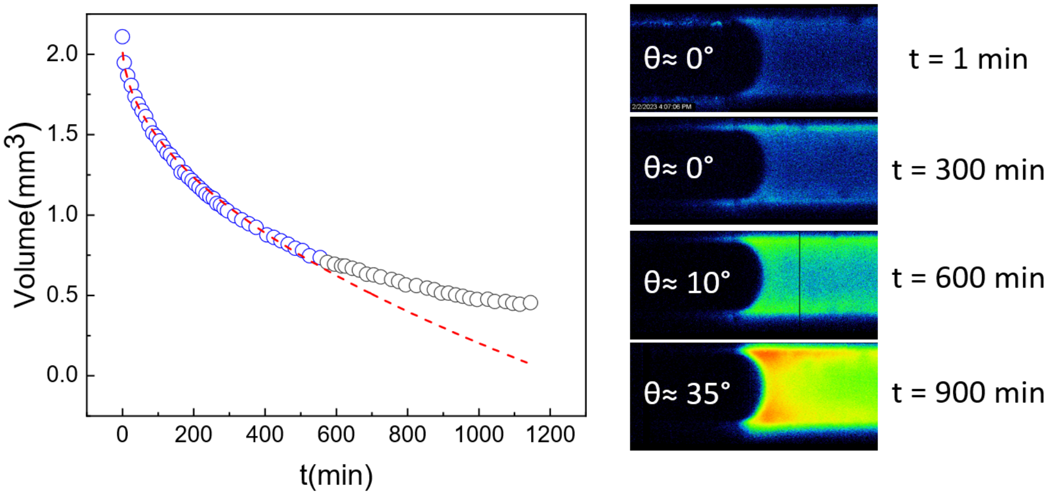

Figure 6: Contact angle evolution during the sol-gel transition of MTS inside a round capillary.

the solution is perfectly wetting the porous media at the beginning of the drying (thin films on the walls) and, after gelation, the contact angle reaches values of roughly 30°. During the gelation, the viscosity of the solution also increases from 0.1 Pa.s to 10^5 Pa.s. The viscosity change is thus largely dominant over the contact angle change.

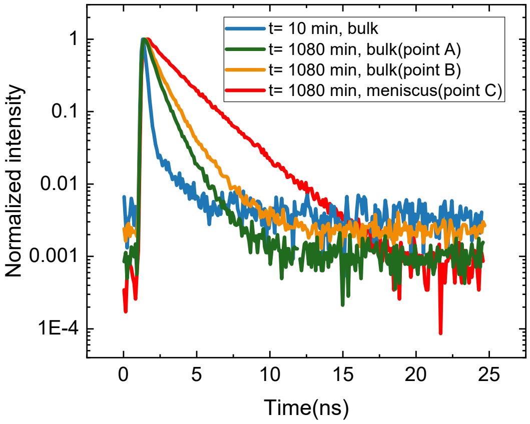

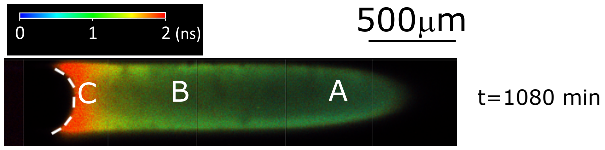

Name	τ1	A1	τ2	A2	<τ>	χ^2
**t= 10 min, bulk**	0.17	25.4	1.52	0.57	0.2	1.12
**t= 1080 min, bulk(point A)**	0.5	14.1	1.17	5.81	0.69	1.054
**t= 1080 min, bulk(point B)**	0.66	9.99	1.46	5.25	0.94	1.095
**t= 1080 min, meniscus(point C)**	1.27	4.23	2.4	9.1	2.048	0.978

Figure 7: Fluorescence decay curves of MTEOS at t=1080 min in a round capillary at various x positions during the Sol-Gel Transition. The blue curve represents the reference decay curve for the bulk solution at t=10 min.

6. Skin formation in round capillary

As described in the main text, the meniscus undergoes recession due to evaporation, leading to the accumulation of macromolecules at its surface. Consequently, a gradient of macromolecules is formed within the capillary.In Fig.S7, the Fluorescence decay curves of MTEOS at t=1080 min in a round capillary at various x positions during the Sol-Gel Transition are shown.

In Fig.S8, the fluorescence lifetime values are plotted against the distance from the surface of the meniscus. Notably, it is evident that the fluorescence lifetime gradient from the meniscus surface to the bulk region intensifies over time.

7. Protocole for the 2D porous media

We designed in this study quasi-2D micromodels of porous media following the protocol detailed in.[2] We recall here the main steps. Rectangular borosilicate microcapillaries (Vitrocom) are cut to the volume $13 \times 3 \times 0.3\text{mm}^3$. One side is melted with a torch and the capillaries are filled with soda lime glass beads of diameter 210-250 μm (Polyscience). To obtain well-packed model porous media, the samples are centrifuged, filled again if necessary

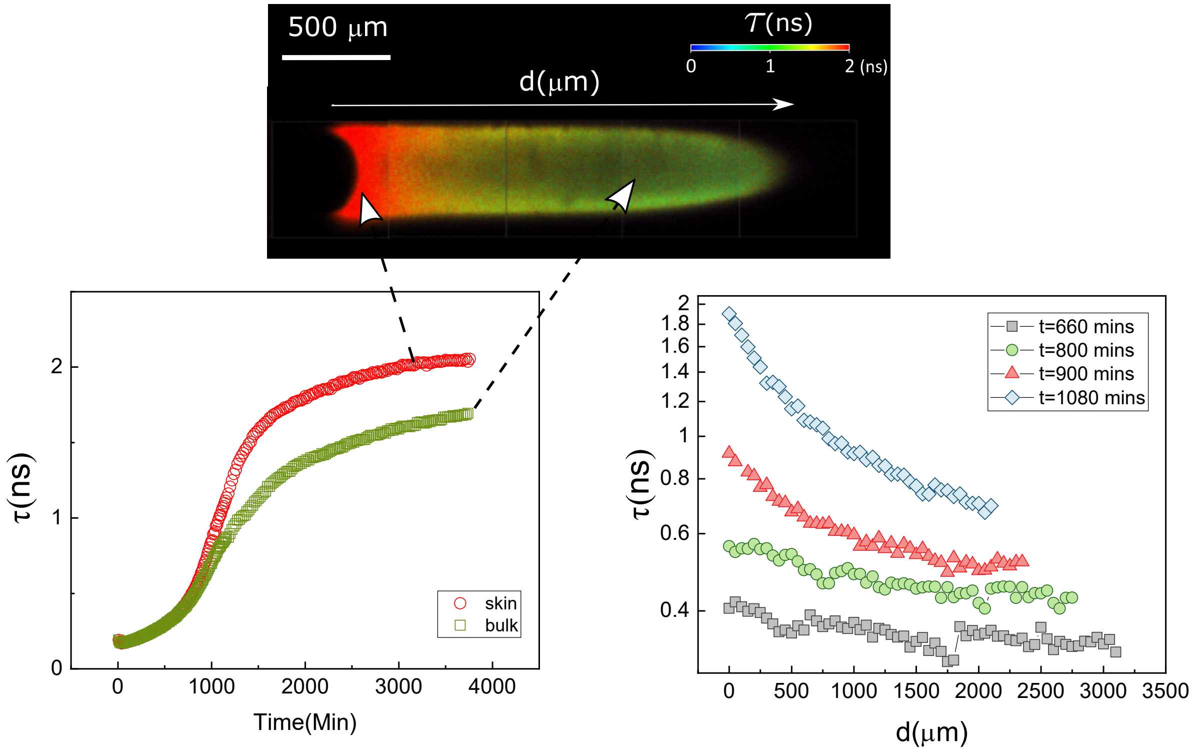

Figure 8: Skin Formation in a Round Capillary. The fluorescence lifetime as a function of time is presented for both the skin layer and bulk region, depicting the evolution of skin formation over time. Additionally, fluorescence lifetime profiles at four distinct time steps are plotted as a function of distance from the meniscus, allowing translation to viscosity as explained in the previous section.

and heated near the glass transition temperature of the glass beads, so that they are sintered together by forming bridges but without deforming their overall shape. Quasi 2D porous media with two pore size distributions (heterogeneous porous media) can the also obtained by mixing the glass beads with NaCl crystals of sizes 150-300 μm prior to the filling of the capillaries. After the heating step, the NaCl crystals (still entrapped in the capillary as their melting point is much higher) are removed by washing several times the sintered porous media with water.

References

(1) Brinker, C. J.; Scherer, G. W. *Sol-Gel Science: The Physics and Chemistry of Sol-Gel Processing*; Academic Press, 1990.

(2) Liefferink, R. W.; Naillon, A.; Bonn, D.; Prat, M.; Shahidzadeh, N. Single layer porous media with entrapped minerals for microscale studies of multiphase flow. *Lab on a Chip* **2018**, *18*, 1094–1104.
